# Translation of genome-wide association study: from genomic signals to biological insights

**DOI:** 10.3389/fgene.2024.1375481

**Published:** 2024-10-03

**Authors:** Winter S. Bruner, Struan F. A. Grant

**Affiliations:** ^1^ Center for Spatial and Functional Genomics, Children’s Hospital of Philadelphia, Philadelphia, PA, United States; ^2^ Division of Human Genetics, Children’s Hospital of Philadelphia, Philadelphia, PA, United States; ^3^ Department of Genetics, Perelman School of Medicine, University of Pennsylvania, Philadelphia, PA, United States; ^4^ Department of Pediatrics, Perelman School of Medicine, University of Pennsylvania, Philadelphia, PA, United States; ^5^ Division of Endocrinology and Diabetes, Children’s Hospital of Philadelphia, Philadelphia, PA, United States

**Keywords:** GWAS, genome-wide association study, snps (single nucleotide polymorphisms), variant to gene, complex traits genetics., function

## Abstract

Since the turn of the 21st century, genome-wide association study (GWAS) have successfully identified genetic signals associated with a myriad of common complex traits and diseases. As we transition from establishing robust genetic associations with diverse phenotypes, the central challenge is now focused on characterizing the underlying functional mechanisms driving these signals. Previous GWAS efforts have revealed multiple variants, each conferring relatively subtle susceptibility, collectively contributing to the pathogenesis of various common diseases. Such variants can further exhibit associations with multiple other traits and differ across ancestries, plus disentangling causal variants from non-causal due to linkage disequilibrium complexities can lead to challenges in drawing direct biological conclusions. Combined with cellular context considerations, such challenges can reduce the capacity to definitively elucidate the biological significance of GWAS signals, limiting the potential to define mechanistic insights. This review will detail current and anticipated approaches for functional interpretation of GWAS signals, both in terms of characterizing the underlying causal variants and the corresponding effector genes.

## Introduction

The pathogenesis of common, complex human traits and diseases emerge as a consequence of the interplay between environmental and genetic factors. To uncover the genetic underpinnings of such traits, studies have successfully employed genome-wide association study (GWAS) to identify susceptibility loci. When a GWAS is conducted, differences in allele frequencies across hundreds of thousands to millions of single nucleotide polymorphisms (SNPs) assayed in one experiment are assessed by comparing the genotypes of individuals with and without a trait of interest (dichotomous), such as asthma, or treating the trait as a continuous variable (quantitative), such as body mass index. One can identify loci associated with a specific disease or trait of interest by evaluating allelic frequency differences that remain statistically significant after correcting for the large degree of multiple comparisons across the genome. This method crucially relies on linkage disequilibrium (LD) to inform the analysis, which can readily aid in identifying associated genetic loci; however, this same factor can limit implications of the actual underlying causal functional variants driving the pathogenesis of the phenotype of interest. For this reason, various tools and study designs have been leveraged to carry out GWAS follow-up studies to uncover which variants are casual for complex traits, along with implicating the corresponding effector genes.

Although GWAS has proven successful in uncovering trait-associated genetic susceptibility loci, ranging from breast cancer to migraine to type 2 diabetes (Bradfield et al., 2012; Bradfield et al., 2019; Easton et al., 2007; Papaemmanuil et al., 2009; Xue et al., 2018; Genome-wide association study identifies new, 2009), there are associated challenges with the overall study design. The ability to obtain statistical power of 80% or more for genetic associations stems from the ability to recruit a sufficient sample size for the GWAS study, which can often prove challenging (more information on GWAS sample size and cohort-based replication studies can be found elsewhere) (Uffelmann et al., 2021). Low sample size and its impact on statistical power contributes to type I and II errors, directly and negatively impacting downstream follow-up studies (Serdar et al., 2021; Banerjee et al., 2009; Krzywinski and Altman, 2013) Typically, collaboration is required to meet such high demands for appropriate sample sizes for statistical power and allowing for the opportunity to replicate initial findings within independent datasets. Additionally, large-scale collaboration efforts lend themselves to subgroup analysis, allowing for additional investigation of complex diseases and traits. With independent and worldwide genomic data collection sites, incorporating different ancestral data collectively can be accomplished through trans-ethnic meta-analysis. Further subgroup analysis can be accomplished based on age, sex, or other dichotomous characteristics to find novel loci for further function follow-up studies. This has been successfully carried out in complex diseases or disorders such as childhood obesity (Bradfield et al., 2012; Bradfield et al., 2019), body mass index (Akiyama et al., 2017), migraines (Anttila et al., 2013), to name a few.

Additionally, GWAS has to account for population-biased findings. Since the allele frequencies used for comparisons often originate from European ancestry, findings from GWAS efforts often need to be more representative across various ancestral groups, resulting in replication challenges across populations (Peterson et al., 2019). As such, combined with remaining power challenges, GWAS is still limited in addressing a large portion of the ‘missing heritability (Manolio et al., 2009; Matthews and Turkheimer, 2022) for common complex traits. Furthermore, GWAS are often performed with SNP array data heavily biased towards common variants (MAF ≥5%) (Momozawa and Mizukami, 2021; Gibson, 2012). This subsequently limits the potential findings of casual rare variants (MAF <1%) (Momozawa and Mizukami, 2021; Gibson, 2012; Wainschtein et al., 2022). As more studies include increasingly larger sample sizes from diverse ancestry and include better imputation panels, the degree of missing heritability remaining to be characterized should narrow (Momozawa and Mizukami, 2021; Gibson, 2012; Wainschtein et al., 2022).

The results of a GWAS are also limited to simply detecting genetic signals. Indeed, such signals themselves cannot pinpoint the true causal variant (s) in LD with the SNP producing the overall lowest *P*-value. This means that the causal variant is not typically assayed directly in the given genotyping assay. Additionally, given the usual polygenicity of common complex traits, the magnitude of each signal is relatively small, with only the additive effects of loci driving the overall genetic etiology of the phenotype of interest. Furthermore, genetic effects are often cell-type specific. As such, determining which cell or tissue type is impacted by GWAS loci has often proven arduous. Together with phenotype heterogeneity, these features of GWAS make mechanistic follow-up analyses challenging.

Although multiple examples of GWAS functionalization attempts exist, one of the most noteworthy examples is at the *FTO* obesity locus (Frayling et al., 2007). This very robust association signal located within an intronic region of the *FTO* gene has been widely replicated across different studies involving different ethnicities (Hassanein et al., 2010; Okada et al., 2012; Wen et al., 2012; Loos and Yeo, 2014) and age groups (Bradfield et al., 2012; Bradfield et al., 2019; Grant et al., 2008; Felix et al., 2016; Elks et al., 2010; Elks et al., 2012). Although there are hundreds of studies validating this association signal with obesity risk, it is becoming clear that the *FTO* gene itself may not be the causal effector gene at this key associated signal. Research assessing the genomic interactions at this locus found a direct contact between the *FTO* intronic region harboring the genetic signal and the *IRX3* gene (Smemo et al., 2014). This led to the conclusion that there is an enhancer imbedded within the *FTO* intron directly influencing the regulation of the neighboring *IRX3* gene (Smemo et al., 2014). Additional work in primary adipocytes further showed that knockout of *IRX3,* together with the next gene along *IRX5*, directly impacted thermogenetic properties and, by extension, demonstrated their role in obesity (Claussnitzer et al., 2015). This is a key example of why GWAS follow-up studies can be time-consuming. Despite such difficulties, establishing casual genetic influences from a GWAS is attainable, especially when incorporating available public resources and cutting-edge techniques (discussed below) to enable important follow-up study designs to reveal crucial novel biological insights.

**FIGURE 1:**
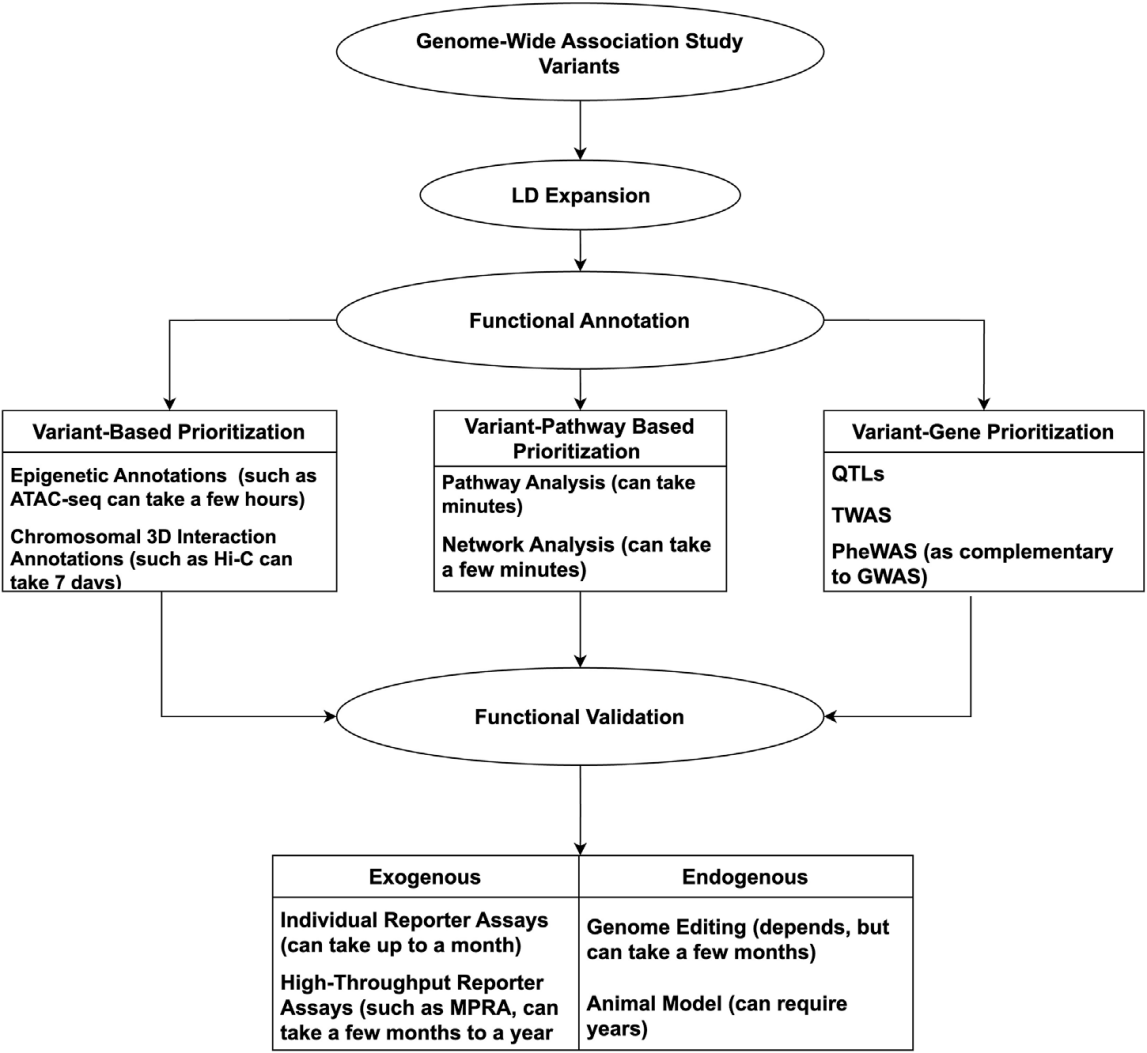
Overview of methods used for functional validation of GWAS variants.

Considering the complexities of GWAS, from the plethora of data to the underlying complex gene-gene/gene-environment interactions, following up on potential leads can appear daunting. Computational tools and technologies can be incorporated to offset such constraints, having proven successful and timely. With so many advances, it is timely to review the available resources to conduct a GWAS mechanistic ‘variant-to-function’ (V2F) follow-up successfully. As such, we will highlight methods and techniques involved in GWAS V2F studies, emphasizing more recent high-throughput methods. An overview of the discussed tools and techniques used for GWAS follow-up can be seen in Figure 1.

### Genetic signal follow-up strategies

Non-coding variants represent over 90% of GWAS reports, which is a large contributing factor to why GWAS follow-up can be so arduous (Schipper and Posthuma, 2022). Although variants can also be found within a gene coding region it potentially regulates, it is still imperative to consider variants in LD that may still reside in non-coding regions (McCarthy et al., 2008). Challenges in understanding the genotype to phenotype relationship resulting from the putative regulation of a noncoding variant, requires connection between an associated variant to gene(s) regulation, and by extension tissue site(s) of mechanistic action. Based on the association alone, researchers cannot determine which variants are causal, the putative gene effector target(s), or tissue-specific involvement. This is where incorporating previously generated data, such as fine-mapping, functional annotations, and a combination with multi-omics approaches plays a role in elucidating the overall biological underpinnings from a GWAS-nominated variant. Given that variant-to-gene methods need to be conducted in a human setting in the first instance, animal models can only be leveraged subsequently, albeit often successfully, once such leads are determined (Palermo et al., 2023; Soleimanpour et al., 2014; Srivastava et al., 2019).

### Variant/gene prioritization approaches and tools

With the myriad of signals discovered by GWAS, narrowing down variants with a higher probability of being causal becomes necessary. Signals detected by GWAS typically do not necessarily represent the causal variant for a given phenotype, but rather represent a tag-SNP in LD with the underlying causal variant(s). Indeed, understanding the underlying LD structure is the initial step in making sense of GWAS signals. Comprehensively assessing both tag-SNPs and their LD proxies facilitates the acquisition of the true causal variant(s) in any functional follow-up approaches (Schaid et al., 2018; Raychaudhuri, 2011). This can be achieved by incorporating fine-mapping into the study design to aid in the prioritization of candidate causal variants by considering both LD patterns and association statistics.

Fine-mapping helps narrow down a list of GWAS signals through a combination of statistical approaches, in conjunction with functional annotations. Types of statistical models used for fine-mapping include Bayesian-based methods (Schaid et al., 2018; de los Campos et al., 2023), heuristic (Schaid et al., 2018), penalized regression (Schaid et al., 2018), and conditional association analysis (Kocarnik et al., 2018). When wanting to obtain more information about more than one SNP collectively producing an effect, the Bayesian method may prove most beneficial (Schaid et al., 2018). Many approaches are available that include the Bayesian-based method for fine-mapping, such as CARMA (Yang et al., 2023a), SUSIE (Wang et al., 2020), PAINTOR (Kichaev et al., 2014), CAVIAR (Wang et al., 2020), etc. Following fine-mapping efforts, an assessment of the genomic landscape can be conducted to determine which variants reside in genomic regions areas which accessible and potentially functional. This can be accomplished through various annotation methods described below.

Work was recently conducted in Alzheimer’s Disease (AD) incorporating variant prioritization via three fine-mapping approaches (Bayesian, FINEMAP, and PAINTOR) in conjunction with annotation-based data from primary microglia and iPSC-derived macrophages (Schwartzentruber et al., 2021). This work revealed 21 variants being prioritized as most probable (>50%) for causality, and an additional 79 variants within the 10%–50% potential variants of interest (Schwartzentruber et al., 2021). Some prioritized SNPs were close to already established AD genes, like *BIN1(43)*. In addition to these known genes, there were new AD risk genes uncovered through leveraging fine-mapped SNPs (Schwartzentruber et al., 2021). Such variant-to-gene pairs included rs143080277 in *NCK2*, rs2830489 near *ADAMTS1*, and rs268120 in *SPRED2(43)*. Similarly, fine-mapping-based work in chronic lymphocytic leukemia (CLL) led to successful GWAS V2F follow-up (Slager et al., 2013). Using genotype data from over 2000 participants, fine-mapping was conducted (Slager et al., 2013). Results revealed a functional connection between rs1044873 and the *IRF8* gene (Slager et al., 2013).

### Functional annotation methods

#### Epigenetic and chromosomal-based techniques

Variant annotation methods that incorporate epigenetic data have become a standard approach to elucidating the functional consequences of genetic variants. Assessment of DNA accessibly can be caried out in multiple cell types to reveal underlying gene regulatory roles of chromatin organization in a given cell type, which in turn suggests how such activity confers its trait effects. Integrating various publicly available resources can further aid the prioritization of GWAS-identified variants. Assay for transposase-accessible chromatin sequencing (ATAC-seq) (Buenrostro et al., 2015) has proven to be an efficacious assay in assessing GWAS variants and functionality for complex diseases. A study focusing on type 2 diabetes (T2D) determined that the open chromatin landscape in human pancreatic islets cells differed between samples obtained from individuals with and without T2D (46). A total of 13 T2D associated SNPs were found in regions marked within open chromatin accessibility sites which were located near genes such as *TCF7L2, ADCY5*, and *GCH* (Bysani et al., 2019). In addition to the 13 SNPs, there were 67 SNPs that were in LD with T2D associated SNPs and further annotated to known T2D genes (such as *PPARG*, *FTO*, and *KCNJ11*) (Bysani et al., 2019). Another study focused on T2D used a combination of pancreatic cell expression data, chromatin accessibility, and network analysis methods to prioritize the gene *RFX6* from GWAS results for the disease (Walker et al., 2023). This study took a different approach for incorporating ATAC-seq by using this technique to assess the chromatin architecture following the knockdown of the putative T2D causal gene (Walker et al., 2023). Knockdown of *RFX6* in beta-pancreatic cells not only resulted in substantial variation in gene expression, but also provided evidence of dysregulation of regulatory elements harboring T2D GWAS variants via changes in genome-wide chromatin states (Walker et al., 2023).

As such, these annotations help determine a variant’s context with respect to a typically non-coding role as a potential regulatory element. Identifying whether a genetic variant resides in a regulatory element (such as promoter, enhancer, or transcription binding site) can be highly informative when determining subsequent biological consequences with respect to gene expression. Furthermore, assessing epigenetic markers in a cell type-dependent manner can help determine gene regulation specificity, adding important context to cell and tissue involvement for a given complex trait or disease. A popular resource for assessing variant annotations includes the Encyclopedia of DNA Elements (ENCODE) Consortium, which consists of histone modification, expression, and chromatin conformation data across different cell types (Luo et al., 2020).

Histone modifications are an essential aspect of gene regulation by influencing how tightly or loosely DNA is packaged, which indicates areas of the genome that are accessible and available for gene transcription. Acetylation and methylation patterns are histone modifications often leveraged to assess poised or inactive cell-specific chromatin states (Karlić et al., 2010). Histone marks used to determine overall DNA accessibility help determine whether a specific region is accessible to transcriptions factor (TF) binding, and therefore a potentially functional element. Regions with histone marks like H3K4me3 and H3K27ac are typically defined as promoter/open chromatin regions (Barski et al., 2007; Creyghton et al., 2010), while marks like H3K27me3 indicate closed chromatin states (Cai et al., 2021). Such histone modification patterns can be assessed by a number of available techniques, which are explored in greater detail elsewhere (Mansisidor and Risca, 2022). Examples of some techniques used to assess chromatin accessibility include not only ATAC-seq (discussed in previous paragraph), but also formaldehyde-Assisted Isolation of Regulatory Elements sequencing (FAIRE-seq) (Giresi et al., 2007), DNase I hypersensitive sites sequencing (DNase-seq) (Song and Crawford, 2010) and sequencing of micrococcal nuclease sensitive sites (MNase-seq) (Deng et al., 2022; Wong et al., 2023). Although many options are available, subtle nuances between the available techniques make some techniques more appealing than others depending on the specific application. Regarding experimental time and output success among the available options, ATAC-seq has become a gold standard, with an approximate three-hour protocol for preparation (Buenrostro et al., 2015; Grandi et al., 2022), where transposases are used with DNA-associated adaptors for subsequent high-throughput sequencing (Buenrostro et al., 2015).

Another significant component of the genomic landscape to consider is chromatin 3D interactions, which offer insight into physical connections between GWAS-implicated candidate causal variants and putative effector genes. Understanding which gene(s) are regulated by non-coding GWAS associated variants is essential to understanding complex traits fully. This is where the power of incorporating chromosomal capture techniques become apparent.

Chromosomal capture techniques involve crosslinking interacting genomic loci to each other, followed by high throughput sequencing (Dixon et al., 2012). The sequencing results generate a map of interacting segments from the genome, which can help nominate candidate effector genes controlled by regulatory elements harboring GWAS associated variants. Various techniques have been developed that can be used for such a purpose, and indeed TADs can be defined with chromosomal capture techniques (Zufferey et al., 2022).

Inclusion of chromosomal conformation capture techniques to determine gene regulation by GWAS loci has been successful in a multitude of studies involving complex diseases and traits. One study identified 38 candidate genes potentially involved in obesity by incorporating promoter Capture-Hi-C techniques within primary adipocytes (Pan et al., 2018). Following promoter Capture-HiC, the study implicated interaction with GWAS SNPs and three additional genes (rs8076131 to *ORMDL3*, rs1017546 and rs3784671 with *LACTB*, and rs10774569 with *ACADS*) (Pan et al., 2018). Use of related techniques led to the discovery of inflammatory bowel syndrome (IBD) target genes that were regulated by 92 regulatory elements previously associated with IBD (Meddens et al., 2016). Our study on systemic lupus erythematosus (SLE) evaluated putative target genes of GWAS signals by combining follicular helper T cells (TFH) (cells with SLE involvement), open chromatin sites, and three-dimensional genomic architecture as defined by Capture C (63). Incorporation of Capture C implicated genes *BCL6* and *CXCR5*, which were previously identified as TFH regulators (Su et al., 2020). The study further assessed the putative target genes via CRISPR/CAS9 genome editing techniques, which revealed key genes important in regulating crucial cytokine involvment in B cell antibody production (Su et al., 2020). Such examples show the power of integrating chromosomal conformation techniques to determine targeted genes by non-coding variants and can lead to a more comprehensive understanding of the genomics driving specific complex traits or diseases.

Studies involving chromatin 3D interactions and genome organization have found regions with increased frequency of contact referred to as topologically associated domains (TADs) (McArthur and Capra, 2021). TADs provide a spatial framework for the genome while facilitating proper gene regulation (McArthur and Capra, 2021), and has been used by investigators to define the search space for defining an underlying effector gene at a given GWAS locus. Variants within noncoding regulatory elements, such as enhancers, localize within three dimensional TADs that are readily assessed through various chromosomal capture methods that asses chromatin 3D interactions (Dixon et al., 2012; McArthur and Capra, 2021). An overview of popular and useful resources for chromatin accessibility data integration can be found in Table 1.

**TABLE 1:** Resource examples used in the GWAS V2F follow-up.

Resource	Data access/Usage
Functional ANnoTation Of the Mammalian genome (FANTOM) (Lizio et al., 2019; Lizio et al., 2015)	Project FANTOM is committed to producing an atlas of human transcripts and is currently on its 6th iteration with a focus on long noncoding RNAs (lncRNAs) (Ramilowski et al., 2020). Previous FANTOM5 work focused on collecting transcriptome data of cells under different states. Data includes transcriptomics of different cell types over various time course and exposures (Noguchi et al., 2017). https://fantom.gsc.riken.jp/
ENCODE Consortium (Luo et al., 2020)	Available annotations include open chromatin, histone marks, transcription factor binding, gene expression, transcription start site, RNA binding protein occupancy, DNA methylation, 3D chromatin interactions, and topologically associated domains data. https://www.encodeproject.org
Roadmap Epigenomics Mapping Centers (REMC) (Kundaje et al., 2015)	DNase, DNAme, histone modification, and RNA-seq data from relatively normal human cell and tissue samples. https://egg2.wustl.edu/roadmap/web_portal/index.html
International Human Epigenome Consortium (IHEC) (Stunnenberg and Hirst, 2016)	Epigenetic profiles of both diseased and unremarkable states with collected mRNA-seq, DNAme, WGBS, and histone modification data. Data collected has originated from worldwide projects and include BLUEPRINT, CEEHRC Epigenomic Platform Project, CREST, and DEEP. https://epigenomesportal.ca/ihec/
Human Genome Browser (Kent et al., 2002)	Web-based tool that allows integration of annotated data across entire genomes to more readily visualize. https://genome.ucsc.edu
Galaxy (Galaxy Community, 2022)	Bioinformatics web-based platform with many computational tools for analysis for a variety of reason (e.g., RNA-seq, ChiP-seq, variant calling, etc.) Allows users to upload own data to analyze, as well as incorporate publicly and shared available data. Important to understand local legal guidelines, especially when analyzing human genomic datasets on a web-based platform. https://usegalaxy.org
Open Targets Genetics (Ghoussaini et al., 2021; Mountjoy et al., 2021)	Web-based platform that integrates GWAS, various QTL, Hi-C, and DNase Hypersensitivity Sites to aid in causal variant and gene target prioritization. https://genetics.opentargets.org/
Genotype-Tissue Expression project (GTEx) (The Genotype-Tissue Expression GTEx project, 2013)	Both RNA-seq and genotype data is used to provide information on human gene expression by tissue. https://www.gtexportal.org/home/

Chromosomal capture techniques including Capture-C (65), ChIA-Pet (Han et al., 2018), Hi-C (59,65), chromosome conformation capture carbon copy (5C) (Dixon et al., 2012; Han et al., 2018; Dostie et al., 2006), chromosome conformation capture-on-chip (4C) (Dixon et al., 2012; Han et al., 2018; Simonis et al., 2006), and other chromosome conformation capture (3C) (Han et al., 2018) techniques are all methods that are available for genomic interaction assessment purposes (Luo et al., 2020; Karlić et al., 2010). Although there are some differences among chromosomal capture technique methods, each method includes four main steps (crosslinking, fragmenting, ligating, and sequencing steps). Each approach requires an initial step for crosslinking chromatin. The genome is subsequently fragmented with endonucleases and then ligated. The ligation step is used to join the interacting genetic loci to each other, which is reverse crosslinked in preparation for sequencing or quantitative polymerase chain reaction (PCR) to identify genomic interactions (Dostie et al., 2006; Simonis et al., 2006; Naumova et al., 2012; Lieberman-Aiden et al., 2009; Fullwood et al., 2009; Li et al., 2010; Zhao et al., 2019a; Fullwood and Ruan, 2009).

#### Quantitative trait loci (QTL)

QTL analysis is another method that can aid in prioritizing variants that are involved in a disease or trait. A QTL can be used to focus on a genomic region associated with a phenotype or trait, which is beneficial when attempting to implicate a gene for GWAS V2F follow-up (Powder, 2020). QTLs will typically incorporate marker and quantitative data to connect observed trait variation to genetic variations within a given population (Powder, 2020). Various types of QTL analysis exist and rely on various combinations of genotype, RNA-seq, chromatin accessibility, and/or methylation data (Powder, 2020). QTL-based data is based on observable, quantitative traits motivated by answering the question of how genetic variation impacts said trait. QTLs can further be used to assess the influence of genetic variation diseases and response to various treatments. Some of the more commonly used QTL analyses include expression quantitative trait loci (eQTLs) (Porcu et al., 2019), protein quantitative trait loci (pQTLs) (Xu et al., 2023), and chromatin accessibility quantitative trait loci (caQTLs) (Khetan et al., 2021; Kumasaka et al., 2019).

The more well-known type of QTL is the eQTL, which is used to determine how a specific genome region impacts gene expression variation. In order to conduct an eQTL study, both genotype and RNA expression data are required. Combining such data for eQTLs can aid in the GWAS V2F follow-up process to determine which genes are influenced by genetic differences in complex diseases or traits. Studies that have overlapped eQTL and GWAS data have been able to address the link between GWAS signals and potential target genes through ‘co-localization’. One example was seen in the context of an AD study, where combined eQTL and mQTL data was used to prioritize SNPs and then to connect them to putative target effector genes (Zhao et al., 2019b). Findings from the study indicated an association between 653 SNPs and 25 genes, with 93 of the SNPs being significant for both eQTL and mQTL data (Zhao et al., 2019b). Furthermore, 10 out of the 25 genes found were previously identified in the literature as already being involved in the genetic etiology of AD (79). Another study used eQTL to assess immune related diseases and nominate 127 candidate disease genes following colocalization of eQTL and GWAS associated SNPs across 11 different diseases (Soskic et al., 2022). The eQTL data was generated from 119 human derived isolated and activated naive and memory CD4^+^ T-cell. Each cell was profiled at resting, 16 h, 40 h, and 5 days (Soskic et al., 2022). The eQTL and GWAS SNP results implicated genes in a broad range of immune related diseases, which included Crohn’s disease, multiple sclerosis and SLE (80). More details on various types of QTL/GWAS colocalization and methods can be found elsewhere (Hormozdiari et al., 2014; Kang et al., 2023; Zuber et al., 2022; Cano-Gamez and Trynka, 2020; Suhre et al., 2021; Zhang et al., 2024; Abood and Farber, 2021; Fabo and Khavari, 2023).

Similar to eQTLs, pQTL and caQTL have major applications. Although these approaches serve slightly different purposes, they incorporate genotype data with protein levels for pQTLs, and chromatin accessibility for caQTLs. A previous study focusing on serum protein successfully overlapped pQTLs with lead GWAS variants for multiple different phenotypes (Gudjonsson et al., 2022). In the study, two body mass index (BMI) associated loci were faound to overlap with protein serum levels of Agouti signaling protein (ASIP) (Gudjonsson et al., 2022). The study also found waist-to-hip ratio GWAS signals within the *LRRC36* gene overlapping with levels of Agouti-related protein (Gudjonsson et al., 2022). One caQTL study had success when assessing GWAS signals for T2D (77). Chromatin accessibility sites were assessed in pancreatic islet cells, resulting in the nomination of causal variants at 13 GWAS loci (Khetan et al., 2021). These candidate causal loci were then functionally assessed *in vitro* via luciferase assay in MIN6 cells (Khetan et al., 2021). Out of the 13 loci, more than half were identified as having differential allelic regulatory roles (Khetan et al., 2021).

Additional QTL approaches that can be applied to GWAS follow-up are outlined in Table 2.

**TABLE 2:** List of various QTL resources available with additional information on the data required to perform each type of QTL and what question each type of QTL can answer.

Gene expression stage	QTL type	Abbreviations	Utility for incorporation into study design
Epigenetic Regulation	Methylation QTL	meQTL (Villicaña and Bell, 2021) mQTL (Lyu et al., 2021) methQTL (Scherer et al., 2021)	Genome-wide genotype in addition to epigenetic/histone marker data (methylation and/or acetylation) can be used to distinguish variant vs. epigenetic impact on trait of interest. Such data is also incorporated to identify associations between variant(s) and chromatin state (open or closed)
	Histone QTL	hQTL (Zheng et al., 2020)	
	Chromatin Accessibility QTL	caQTL (Zheng et al., 2020)	
	Transcription factor binding QTL	tfQTL (Watt et al., 2021) bQTL (Tehranchi et al., 2016)	Determining potential genomic regions involved in gene regulation in a cell specific manner
Transcriptional Regulation	Expression QTL	eQTL (Zhang and Zhao, 2023)	Genome wide genotype data and gene expression data (RNA-seq) to determine how variants impact gene expression of gene(s)
Post-Transcriptional Regulation	RNA Editing QTL	reQTL (Zheng et al., 2020) edQTL (Park et al., 2021)	When assessing the impact of genetic variation on protein levels. This can be due to underlying involvement in regulation involving pre-mRNA regulation by variation in capping, splicing, or polyadenylation
	microRNA QTL	miRQTL (Huan et al., 2015)	
	Splicing QTL	sQTL (Zheng et al., 2020)	
	Competing Endogenous RNA QTL	cerQTL (Zheng et al., 2020)	
	Alternative Polyadenylation QTL	apaQTL (Li et al., 2023b)	
Translational Regulation	Ribosome Occupancy QTL	riboQTL (Zheng et al., 2020) rQTL (Battle et al., 2015) roQTL (Cenik et al., 2015)	Ribosomal occupancy assessment can aid in determining potential underlying translational efficiency impact from putative variants (Ozadam et al., 2023)
	Protein Expression QTL	pQTL (Zheng et al., 2020)	GWAS/WES/WGS data combined with protein-based data to link variants to protein quantity (Xu et al., 2023)
Post-Translational Regulation	Metabolic QTL	metaQTL (Zheng et al., 2020)	Determine whether allelic differences explain differences in metabolite levels (Carreno-Quintero et al., 2012)

#### Transcriptome-wide association study (TWAS)

Similar to QTL, Transcriptome-Wide Association Study (TWAS) (and the newer casual TWAS that allows for confounding adjustments within the model (Zhao et al., 2024)) can aid in gene prioritization for GWAS follow-up. TWAS leverages genomic and transcriptomic data to discover how genetic differences might impact gene expression across different tissues. This approach can further explain how genetic variants found through GWAS not only affect gene expression, but also influence disease risk. A study conducted by Gusev et al. applied TWAS by using expression data across multiple tissues in conjunction with GWAS summary stats focused on traits such as height, body mass index (BMI), and lipids (Gusev et al., 2016). By leveraging this type of data, the study revealed 69 novel gene-trait associations (Gusev et al., 2016).

While many TWAS methods tend to be univariate (such as PMR-Egger (Yuan et al., 2020), PrediXcan (Gamazon et al., 2015), and FUSION(90)), more recent TWAS methods have tried to expand this type of model with the rationale of potential pleiotropic effects (Liu et al., 2021; Feng et al., 2021). One such study that developed a TWAS method referred to as moPMR-Egger which accommodates the analysis of multiple traits at a time (as opposed to one trait), lead to 13.5% increased gene associations findings when applied to United Kingdom biobank traits (Liu et al., 2021). While has different models available, it can still be a great genomic-based tool that offers a potential resolution to determining how GWAS identified variants might function within a biological setting by suggesting the genetic target(s) of GWAS varaints. More in depth information of the various TWAS approaches along with valuable resources can be found elsewhere (Feng et al., 2021; Li and Ritchie, 2021; Zhu and Zhou, 2021; Mashhour et al., 2024; Xie et al., 2021).

While outside the scope of this review, it is worth mentioning the use of Phenome-wide association studies (PheWAS) for its ability to also connect gene-trait associations. A general overview on PheWAS and some resources can be found elsewhere (Liu and Crawford, 2022; Bastarache et al., 2022).

#### Pathway and network analysis

Successful incorporation of pathway and network-based analyses can inform V2F by implicating potential hubs influencing a phenotype of interest. One AD study identified 32 additional candidate genes by including a network analysis in their study (Schwartzentruber et al., 2021). They first generated an overall gene interaction list based on information retrieved from the STRING, IntAct, and BioGRID databases (Schwartzentruber et al., 2021). Through propagation methods utilizing genes found in the initial retrieval process, the study was able to highlight previously established genes associated with AD (Schwartzentruber et al., 2021). Interestingly, the study included high ranked genes for AD GWAS loci with the lowest *P*-values, indicating larger GWAS may be required to fully evaluate all putative loci (Schwartzentruber et al., 2021). Another recent study focused on kidney renal clear cell carcinoma (KIRC) utilized a Protein-Protein Interaction (PPI) network method and the authors elected to further evaluate the top four genes (referred to as “hub” genes) in the associated list (Ali et al., 2023). Through further assessment of their implicated hub genes, the study reported that two out of the four genes were upregulated while the other two were downregulated in KIRC patients (Ali et al., 2023).

Although there is debate regarding the utility and reproducibility of pathway and network based analyses (Tomczak et al., 2018), a wide-range of studies have included such results as a means to further understand potential underlying gene interactions on a broader scale (Yu et al., 2019; Jin et al., 2022). Pathway analysis can be accomplished through either a non-topology or a topology-driven method. A non-topology-driven method is traditionally known as an overrepresentation analysis (ORA) or functional enrichment analysis. An enrichment analysis considers a list of significantly differentially expressed genes (DEG) from a larger given data set. The list is then used to determine the percent of DEG present within a pathway. When more than 10% of DEGs are present in a given pathway, that specific network is considered “enriched” and worth further investigation. ORA tools include FuncAssociate (Berriz et al., 2009), GeneMerge (Castillo-Davis and Hartl, 2003), EASE (Hosack et al., 2003), g:Profiler (Kolberg et al., 2023), DAVID ((Huang et al., 2009a), (Huang et al., 2009b)), WebGestalt (Liao et al., 2019), AmiGO 2 (Carbon et al., 2009), GeneWeaver (Baker et al., 2012), BiNGO (Maere et al., 2005), GoMiner (Zeeberg et al., 2003), ontologizer (Bauer et al., 2008), etc. Functional class scoring (FCS) is another type of ORA similar to functional enrichment analysis but utilizes the entire gene set data instead of only the DEG. Common FCS approaches include GSEA (Subramanian et al., 2005; Mootha et al., 2003), GSA (Mooney and Wilmot, 2015), GlobalTest (Hulsegge et al., 2009), PADOG (Tarca et al., 2012), SAM-GS (Dinu et al., 2007), FunCluster (Henegar et al., 2006), etc.

Gene Ontology (GO) analysis is another ORA-based enrichment method that allows users to assign functional annotations based on various categories. GO analysis can help assign genes or gene products based on molecular, biological, and cellular functions (Zhao et al., 2020). GO analysis is an example of a pathway analysis, which identifies biological pathways involved in a phenotype by using gene expression data and available pathway databases. This approach is often used to help elucidate how genes and variants impact specific cellular pathways. Pathway-based databases can help discern involved gene and protein networks, which helps further understand the underlying interactions within a given complex trait or disease. There are multiple online resources available to incorporate GO analysis. Most notable databases include Kyoto Encyclopedia of Genes and Genomes (KEGG) (Kanehisa and Goto, 2000), PANTHER (Thomas et al., 2022), Reactome (Gillespie et al., 2022), Pathway Commons (Rodchenkov et al., 2020), Wiki-Pathways (Martens et al., 2021), and PathBank (Wishart et al., 2020).

Although non-topology ORA analyses are helpful and can yield biological insights, such analyses do not take into account interactions of genes, which often play a role in contributing to complex disease and traits. For this reason, topology-based approaches can be a better alternative to non-topology ORAs. Pathway topology (PT) considers the independent gene role, position, magnitude, and interactions. Fold change of a gene is propagated onto the gene directly downstream in a pathway. iPathwayGuide (Ahsan and Drăghici, 2017) provides a web-based system to conduct a PT approach to gene expression analysis tools to better rank pathways involved in a given phenotype. More information on pathway analysis and additional resources can be found elsewhere (Maleki et al., 2020).

Pathway analyses provide information regarding gene and gene products within a set pathway impacting a phenotype of interest, but not necessarily how proteins across different pathways potentially interact. Such information can yield additional insight into the underlying biological network leading to a complex disease or trait. For this purpose, PPI networks can help highlight how proteins within various pathways intersect and interact at a given point to serve an overall biological process. A valuable tool for a PPI network includes the STRING (Szklarczyk et al., 2021) database, which can also be used for enrichment and PPI network analysis.

Although it aids understanding of the underlying biological network, the main goal is for functional and mechanistic significance. Insight from additional approaches like phenotype annotation and comparative genomics can further aid in understanding how genetic variants lead to a specific functional outcome (i.e., disease, drug response, biological process, etc.). By incorporating tools like Ensembl (Martin et al., 2023), linking genetic changes to phenotype can help determine functional consequences. Furthermore, using evolutionally conservation data can also help understand functional significance through comparative genomics. Comparing genomic regions across species for GWAS signal interpretation may reveal a conserved gene regulatory site. Using such knowledge can further aid in understanding whether a specific locus is in a region potentially involved in regulating an essential gene in a given biological pathway. Available tools for a comparative genomic approach include VISTA (Frazer et al., 2004; Dubchak et al., 2000), CoGE (Lyons and Freeling, 2008), PipMakers (Schwartz et al., 2000), etc.

### Functional validation methods

#### Individual reporter-based method

Regulatory abilities of putative non-coding regulatory elements are traditionally assessed through individual reporter-based assays. Although reporter assays are not used to explicitly nominate genes being regulated by a putative enhancer region, they can represent an initial step in determining which nominated variants drive expression changes. The luciferase reporter assay system is an example of an individual reporter-based assay that is very useful when investigating the regulation on gene expression. Incorporating reporter assays has shown success in assessing variant regulatory impact of across different complex disease and traits (Zhang et al., 2010; Ustiugova et al., 2019; Rivas et al., 2011; Ramachandran et al., 2022). While investigating opioid addiction and the role of rs569356 in the gene *OPRD1* gene promoter, luciferase reporter plasmids were constructed with the major (A) and minor (G) alleles (Zhang et al., 2010). These constructs were transfected into HEK293 cells, and results indicated the G allele led to an increased expression in the reporter assay (Zhang et al., 2010). The differential allelic response was used to suggest a potential mechanism in regulation of *OPRD1* leading to opioid addition (Zhang et al., 2010). Another study focused on GWAS associated loci in autoimmune diseases, incorporating luciferase reporter assay into their study design to determine allele specific functionality (Ustiugova et al., 2019). Both risk and protective alleles from six associated loci (rs12946510, rs2313430, rs4795397, rs12709365, rs13380815, rs8067378) were included in the reporter assay and assessed in six different cell lines (Nalm6, MP1, Jurkat, MT-2, U-937, and activated U-937) (Ustiugova et al., 2019). The results indicated cell specific differential allelic activity for at least four of the six loci, with the strongest effect seen for rs12946510 across three cell types (Nalm6, MP1, and activated U-937) (Ustiugova et al., 2019).

Although simplistic in design, luciferase assays are still useful in determining whether a specific genomic region harboring GWAS loci potentially impacts gene expression. Luciferase assays use plasmids containing a luciferase reporter gene located downstream of a regulatory element of interest and a minimal promoter (Fan and Wood, 2007). The final reporter construct is transfected into a specific cell type (animal, plant, or bacteria). Since the reporter gene is essentially fused to the regulatory element, detecting transcription changes in the reporter gene is directly correlated to the relative regulatory activity of the regulatory element (cis-acting) (Fan and Wood, 2007). The luciferase gene encodes for a specific enzyme that produces fluorescence, which is quantified by measuring the light intensity (Fan and Wood, 2007).

Although individual reporter assays help determine functional non-coding variants nominated from GWAS, they are very time-consuming. The time constraints associated with individual reporter assays require stringent prioritization of nominated GWAS variants due to their inability to incorporate multiple variants in a single experiment. Current genomic technologies have led to an expanded and comprehensive form of individual reporter-based assays, referred to as high-throughput reporter assays. Such high-throughput reporter assays allows for the evaluation of thousands of putative non-coding regulatory variants simultaneously (Melnikov et al., 2012; Kheradpour et al., 2013; Kwasnieski et al., 2012; White et al., 2013; Inoue and Ahituv, 2015; Arnold et al., 2013). High-throughput reporter assays result in a more streamlined and comprehensive follow-up approach for GWAS-nominated variants.

Most high-throughput reporter assays, like Massively Parallel Reporter Assay (MPRA) and Self-Transcribing Active Regulatory Region Sequencing (STARR-seq) (both discussed below), use a similar plasmid-based concept as utilized in individual reporter assays when determining the relative regulatory ability of specific non-coding regions (Gallego Romero and Lea, 2023; Das et al., 2023). The main difference between individual reporter assays and high-throughput assays is how the expression is quantified. In individual reporter assays, the relative enhancer activity is qualitatively determined using light emission created by substrate-enzymatic reactions (Fan and Wood, 2007; Melnikov et al., 2012; Kheradpour et al., 2013; Tewhey et al., 2016). High-throughput reporter-based assays are far more quantitative by incorporating high throughput sequencing to count generated sequences caused by a specific regulatory region (Gallego Romero and Lea, 2023; Das et al., 2023).

#### High-throughput methods (MPRA)

MPRA is a highly reproducible and sensitive high-throughput reporter assay that simultaneously evaluates thousands of putative regulatory sequences while determining allele-specific activity (Melnikov et al., 2012; Kheradpour et al., 2013; Ulirsch et al., 2016; Lu et al., 2021). Inclusion of the MPRA design has been utilized in a wide range of contexts from answering evolutionary questions (Du et al., 2022) to determining previously unexplored allele specific activity of complex diseases/traits. In fact, multiple studies have shown great success in identifying functional GWAS loci that were previously unexplored through incorporation of MPRA (Matoba et al., 2020; Long et al., 2022; Mouri et al., 2022). One study, in particular, focused on autoimmune diseases where T cell involvement was known. Integrating variants associated with inflammatory bowel syndrome (IBD), multiple sclerosis (MS), type 1 diabetes (T1D), psoriasis and rheumatoid arthritis (RA) were included in the MPRA design and subsequently assessed for their functionality within T-cells (Mouri et al., 2022). With over 18,000 loci assessed, the study found 313 variants with differential expression when comparing the reference and alternative alleles (Mouri et al., 2022). Other studies have achieved success by leveraging data from MPRA and eQTLs combined, allowing further biological perturbations from previously overlooked loci (Choi et al., 2020). This study included over 800 loci associated with melanoma, and assessed their regulatory influence within a melanoma cell line (Choi et al., 2020). Overlapping the MPRA data with local eQTL data, the study could prioritize nine variants for which differential gene expression data appeared to corroborate potential variant endogenous gene regulation (Choi et al., 2020).

Incorporating MPRA to evaluate GWAS-associated variants include constructs with sentinel single nucleotide polymorphisms (SNPs) and variants in LD. Including variants in LD aids in determining whether the SNP directly genotyped for the GWAS is influencing the biological system or the SNP in high LD with the SNP directly genotyped. Once a list of noncoding variants is determined, various manufacturers (such as Agilent Technologies, Dynegene, and Twist Bioscience (Agilent, 2024; Dynegene, 2024; Bioscience, 2024)) can create large-scale oligonucleotide (oligo) library pools. The entire MPRA oligo library is generated or synthesized by microarray technologies and is currently limited to 230 base pairs (bp) (Melnikov et al., 2014). Although traditional MPRAs are highly reproducible and sensitive enough to determine allelic variation, the MPRA reporter plasmid design cannot guarantee biological relevance. The construct may not represent an actual regulatory element since MPRA oligos are limited in sequence length. Regulatory elements often span much larger regions than the length-restricted regions found in MPRA oligos, which may lead to type I and II errors (Blackwood and Kadonaga, 1998). Since regulatory regions can span large regions, it is essential to validate MPRA findings to include genomic regions that span larger bp regions centered on the putative variant that can be transformed into individual reporter plasmids (Blackwood and Kadonaga, 1998). The ability to further evaluate larger regions of any high-confidence regulatory element found in MPRA assays with individual reporter assays is one way to mitigate the negative consequences of the limited sequence lengths in MPRA oligo library pools. Another alternative approach to the limited length of current MPRA designs is the Tiling MPRA method (Ernst et al., 2016). Tiling MPRA allows one to extend the length of testable regulatory regions by using multiple 175bp constructs for one variant loci represented at the center of the construct and varying bp lengths to the left and right from the center of the constructs (Ernst et al., 2016).

In addition to size restrictions, MPRA plasmid pool designs do not consider the endogenous biological context. Although using the same minimal promoter and reporter gene for the entire MPRA library pool is beneficial for direct comparison across individual regulatory elements within the MPRA pool, it lacks biological context and relevance by excluding endogenous promoters and genes. MPRA experiments cannot measure the putative regulatory element’s activity in its endogenous chromatin environment. Additionally, GWAS can nominate noncoding variants that may be involved in gene regulation but do not indicate putative tissue or cell types involved. Lack of cell-specific involvement in a disease or trait becomes another limitation for MPRAs when incorporating library pools for a research design. MPRA libraries are created based on all top GWAS hits, which means false positives and negatives can occur when utilizing this technique within cell types that are not actively involved in the complex trait or disease of interest. Including chromatin accessibility data for specific cell types is one way to avoid such errors when determining which cell type best suits the MPRA design. Alternatively, the high-throughput reporter assay called STARR-seq can supplement the MPRA design to analyze active enhancer regions within a given temporal context (Arnold et al., 2013).

#### High-throughput methods (STARR-Seq)

Similar to MPRAs, STARR-seq can simultaneously determine the regulatory activities of thousands of regulatory elements. Unlike MRPA, STAR-seq method can determine active regulatory regions of the genome in a state/temporal dependent manner while directly assessing cell-specific regulatory activity (Arnold et al., 2013). Studies incorporating STAR-seq design have had success in determining regulatory elements for cellular response to various pharmacological drugs, such as dexamethasone and other glucocorticoids (GC) (Johnson et al., 2018; Penner-Goeke et al., 2023). Such studies placed cells under the influence of steroid-based drugs to induce a GC- response. Regulatory regions that were responsive to the pharmacological insult would reside in open chromatin, allowing for fragmentation and placement into the reporter plasmid for incorporation into the STARR-seq design to detect novel GC-responsive regulatory loci within the genome (Johnson et al., 2018; Penner-Goeke et al., 2023). Incorporating GC-responsive elements into the STARR-seq technique has previously allowed researchers to identify an overlap between functionally validated drug responsive elements that were enriched with identified GWAS variants associated with psychiatric traits (Penner-Goeke et al., 2023). Under the driving idea that a “stress” induced response would result in various psychiatric outcomes, the STARR-seq reporter plasmid was introduced into osteosarcoma and brain glioblastoma cell lines (Penner-Goeke et al., 2023). Furthermore, the identified GC-responsive elements were found to regulate transcripts enriched in genes shown to be differentially expressed in the cerebral cortex of psychiatric disorders, such as schizophrenia (SCZ), major depressive disorder (MDD) and autism spectrum disorder (ASD) (Penner-Goeke et al., 2023).

Importantly, STARR-seq can only include open chromatin accessibility sites in the reporter plasmid pool (Arnold et al., 2013). In some situations, the direct cell-specific assessment makes STARR-seq cost effective compared to MPRA as a plasmid pool is unnecessary for STARR-seq (Das et al., 2023). Instead, any cell type or tissue needed for a project can be used to extract cellular DNA and subsequently sheared to obtain smaller fragments of DNA (typically 300-500bp fragments) (Arnold et al., 2013). The fragmented DNA is transformed into the reporter plasmid. Open chromatin regions can be obtained by targeting regions by incorporating chromatin immunoprecipitation (CHIP) techniques to select for specific transcription factor binding sites or histone modifications associated with open chromatin sites and active enhancers (H3K27ac and H3K4me1) which can be advantageous when only wanting to assess GWAS associated SNP regions (Arnold et al., 2013; Heintzman et al., 2007). The STARR-seq reporter plasmid is then transfected into the cell type from which the regulatory elements were initially derived. Following transfection, the cells are lysed and submitted for high-throughput sequencing (Arnold et al., 2013).

Another difference seen between MPRA and STAR-seq methods is in the subsequent analysis. Variation in the analysis technique is due to the plasmid organization. MPRA plasmids place the regulatory regions upstream of a minimal promoter and reporter gene. The only way to determine regulatory activity in an MPRA experiment is to sequence the unique identifier downstream of the reporter gene. Unlike the MPRA plasmid organization, the STARR-seq method places the regulatory element downstream of the core promoter and reporter gene (Arnold et al., 2013). The basic concept driving the STARR-seq method assumes active enhancers can act independently of their position (Arnold et al., 2013). Placement of the regulatory elements in the STARR-seq plasmid will allow active enhancer regions to transcribe themselves, essentially being their own unique identifier. Analysis for STARR-seq experiments differs from MPRA analysis primarily due to the need for alignment, which is due to the incorporation of a fragmentation step in creating the STAR-seq reporter plasmid pool (Arnold et al., 2013).

#### Genome editing based methods (CRISPR)

Incorporating functional annotation and validation methods can collectively implicate a link between GWAS-nominated variants and target gene expression; however, these techniques still lack the ability to physically confirm whether GWAS loci are causal within an endogenous setting. Recent techniques like CRISPR become useful in fully elucidating how variants impact phenotypes within an endogenous setting. This powerful genome editing technique has shown great utility in a wide range of diseases (such as Parkinson’s (Soldner et al., 2016), schizophrenia (Forrest et al., 2017) and nasopharyngeal carcinoma (Wang et al., 2023)). Furthermore, CRIPSR has been used in pursuit of GWAS loci for complex traits such as bone mineral density (Pippin et al., 2021). Our study focused on bone mineral density connected GWAS associated variants to a target gene by incorporating promoter capture C (172). After obtaining a putative gene target (*EPDR1*), CRIPSR was used to knockdown the gene in a cell model equivalent to mesenchymal stem-cell derived osteoblasts and we showed the gene impacted osteoblast differentiation (Pippin et al., 2021).

CRISPR aids in such investigations by allowing direct quantification of gene expression based on endogenous single nucleotide changes (Wang et al., 2016; Irion et al., 2014). Editing an individual nucleotide in a regulatory sequence allows direct assessment of variant function on putative target gene expression. This was accomplished in a study interesting in bone mineral density and hyperglycemic phenotypes (Sinnott-Armstrong et al., 2021). Chromosomal capture techniques were used to assess *ADCY5* as the potential target of the rs56371916 pleiotropic locus (Sinnott-Armstrong et al., 2021). CRIPSR was implemented to edit the locus in adipose-derived mesenchymal stem cells (AMSCs) to be either homozygous for CC or TT (Sinnott-Armstrong et al., 2021). Following osteoblast or adipocyte induction, *ADCY5* expression was assessed (Sinnott-Armstrong et al., 2021). Findings indicated *ADCY5* expression was increased in osteoblast induction for the TT homozygous genotype, while conversely *ADYC5* expression was decreased in the adipocyte induction for the CC homozygous genotype (Sinnott-Armstrong et al., 2021). Overall, the change in *ADCY5* expression confirmed a pleiotropic effect of rs5637916 seen through lipolysis regulation in adipocytes and lipid-oxidation differentiation of osteoblasts (Sinnott-Armstrong et al., 2021).

Using CRISPR to edit single nucleotide changes requires the Cas9 protein, gRNA, and a repair template strand (Wang et al., 2016; Irion et al., 2014). The repair template strand is used to repair the genomic break caused by the Cas9 protein through the homology-directed repair (HDR) and nonhomologous end joining (NHEJ) mechanisms (Li et al., 2023a). Following nucleotide editing validation, the impact of downstream gene expression can be determined with traditional qPCR techniques. Unfortunately, off-target effects while using CRISPR and designing functional guide RNAs (gRNAs) are a valid concern. Exploiting the high-fidelity use of HDR for single nucleotide editing has the unfortunate drawback of having low efficiency with previous rate estimates of approximately <5% (Wang et al., 2016; Irion et al., 2014; Komor et al., 2017). However, this rate has recently been drastically improved to >50% (Li et al., 2023a).

CRISPR screens can be implemented as a pooled (gRNAs in bulk cells) and arrayed (gRNAs intro CRISPR screens) for a large-scale GWAS follow-up (Bock et al., 2022) or at a single-cell resolution (Faial, 2023). While pooled screens are great for discovery-based research, array-based screens are beneficial for follow-up-based studies. In the context of GWAS signals, array-based CRISPR screens can make implementing single-base pair changes in the genome possible, and measurable phenotypes can be determined based on the perturbation. With the continued use of CRISPR screen applications for GWAS functional follow-up, focus on single-cell CRISPR-based (scCRISPR) applications has increased. Some notable methods that incorporate the scCRISPR approach include CRISP-seq (Jaitin et al., 2016), CROP-seq (Datlinger et al., 2017), Mosaic-seq (Xie et al., 2017), Perturb-seq (Adamson et al., 2016; Dixit et al., 2016), and transcript-informed single-cell CRISPR sequencing (TISCC-seq) (Kim et al., 2024a). Additional details on the progress of CRISPR-based screen approaches has been recently explored by Kim et al. (2024b), Cooper et al. (2024).

#### Artificial intelligence (AI) methods

AI is a general term used to describe technology that utilizes computer algorithms like machine learning or deep learning (Sarker, 2022). The benefits of incorporating AI programs can be seen in their ability to handle large amounts of data, which can significantly decrease the overall time of determining putative gene targets of GWAS variants (or variants in LD with GWAS variants). AI tools have been trained on different datasets for the overall purpose of pattern recognition. Having the ability to assess patterns quickly, AI has become an efficient way to predict which variants might disrupt regulatory motifs and gene function, leading to a more streamline method to prioritize the most relevant variant and putative target gene for functional validation.

Researchers have already benefited from AI integration in tools that can help suggest the potential functional impact of GWAS variants such as the online tools GeneMANIA (Warde-Farley et al., 2010), STRING (Szklarczyk et al., 2021; Martin et al., 2023), PhenoScanner V2 (Kamat et al., 2019), and Variant Effect Predictor (VEP) (Martin et al., 2023; McLaren et al., 2016). Tools like these, capitalize on pattern recognition from intersecting AI and multi-omics datasets (such as transcriptomics, functional annotations, etc.). Other tools, like DeepSEA (Zhou and Troyanskaya, 2015) and ExCAPE (Sturm et al., 2020), use a convolutional neural network (Gu et al., 2018) to determine the potential impact of regulatory elements and gene expression and how this could translate to a pharmaceutical approach, respectively (Zhou and Troyanskaya, 2015; Sturm et al., 2020).

Genetic variants effect predicting tools have further been inhanced by the inclusion of deep learning tool. Some notable examples include ESM (Lin et al., 2023) (iterations include ESM-1, ESM-2, and recently ESM-3), AlphaMissense (Cheng et al., 2023; Tordai et al., 2024), AlphaFold2 (Yang et al., 2023b), and DeepVariant (Chen et al., 2023). Various iterations of ESM focus on model protein structures. ESM assesses evolutionary sequence data and genetic mutations into its model to suggest how variants affect protein folding, stability, and/or interactions (Lin et al., 2023; Callaway, 2024). ESM-3 has extended this protein-based application to predicting new proteins that could be created to enhance current GFP and CRISPR proteins to enhance current techniques used in functional assays (Callaway, 2024). Similarly, AlphaMissense AI tool predicts protein activity caused by variants that impact amino acid sequences by using deep learning and structural bioinformatics (Tordai et al., 2024). Apart from protein prediction effect, other advancements in AI have provided newer tools like DeepVariant that have improved accuracy of mapping variants to genes and/or regulatory elements they might affect (Chen et al., 2023).

Once a target gene or regulatory element is prioritized with the available AI algorithms, other forms of AI tools can further aid in developing the assays used for functional assays. Already mentioned was the latest ESM-3 tool that is actively attempting to create new protein structures based on known sequences that could provide higher efficient CRISPR proteins for genome editing (Callaway, 2024). ESM-3 is currently too new to fully know the degree of efficiency this may provide to current CRISPR techniques used for functional follow-up, but a important to mention in the context of how AI tools may further advance functional assessments in the future (Callaway, 2024). AI-powered design tools like CRISPR-DO provides predictive feedback on designing guide RNAs used for genome editing, which is useful for designing the most efficient gRNA for successful application of CRISPR technique (Ma et al., 2016). Additional AI powered tools and method have been explored elsewhere (Sigala et al., 2023; Nicholls et al., 2020).

## Concluding remarks

Functional work of GWAS signals continue to be an arduous endeavor. Most of the complexity of GWAS follow-up stems from the large percent of predicted variants reside within the noncoding genome. Such nominated variants likely involved in gene regulation, which requires the need to determine target gene(s) and how this may vary across different tissues and under varying stimuli. As technology and computation methods continue to advance, such challenges will steadily decrease (or at least minimize the time to find causal variants and their target gene(s). Continued advancements will further help overcome the additional challenge of answering further questions regarding how GWAS variants might impact gene function within different cells/tissues, cellular state (such as development state), and environmental stimuli (drug exposure, altitude, temperature, etc.).

The future of GWAS signal follow-up is beginning to be positively impacted by the continued interest in artificial intelligence (AI) and machine learning integration. As GWAS data is often too large to follow-up efficiently, technological advancements involving AI promise to quickly assess the most probable GWAS signals responsible for complex diseases and traits. Moreover, an AI-based framework can be incorporated to identify underlying networks of regulation that are currently time-consuming and difficult to explain fully in complex phenotypes. In this manner, AI has the potential to overcome a large portion of the time and financial burden often associated with follow-up studies of GWAS results and further facilitate quicker personalized therapy and medicine.

As genomics and computational approaches continue to advance, most notably with the recent advances in V2F based algorithms such as activity-by-contact (ABC) (Nasser et al., 2021), polygenic priority score (PoPS) (Weeks et al., 2023) and the Effector Index (Liang et al., 2023), GWAS V2F follow-up will become increasingly capable of uncovering the underlying gene-gene and gene-environment interactions that collectively contribute to disease and trait etiologies. Currently, combining multiple techniques for variant prioritization and functional validation methods is an established process for determining causal variants and pathways contributing to complex phenotypes of interest. Most of the methods and techniques lead to large multi-omics and pathway-based datasets that require computational models to dissect the genetic basis of a complex trait comprehensively. Ongoing improvements with high-throughput techniques and computational methods will allow for easier identification of causal genes and pathways than previous capabilities. With the advent and continued advancements of computational algorithms and AI, the inclusion of multi-omics and “big data” will undoubtedly experience a further reduction in the current time restraints required for completing GWAS V2F follow-up.
